# Neurovascular effects of umbilical cord blood-derived stem cells in growth-restricted newborn lambs: UCBCs for perinatal brain injury

**DOI:** 10.1186/s13287-019-1526-0

**Published:** 2020-01-08

**Authors:** Atul Malhotra, Margie Castillo-Melendez, Beth J. Allison, Amy E. Sutherland, Ilias Nitsos, Yen Pham, Courtney A. McDonald, Michael C. Fahey, Graeme R. Polglase, Graham Jenkin, Suzanne L. Miller

**Affiliations:** 1 Monash Newborn, Monash Children’s Hospital, 246 Clayton Road, Clayton, Melbourne, VIC 3168 Australia; 2 Department of Paediatrics, Monash University, Melbourne, Australia; 3 The Ritchie Centre, Hudson Institute of Medical Research, Melbourne, Australia; 4 Department of Obstetrics and Gynaecology, Monash University, Melbourne, Australia

**Keywords:** Brain injury, Intrauterine growth restriction, IUGR, FGR, Preterm, Ventilation

## Abstract

**Background:**

Neonatal ventilation exacerbates brain injury in lambs with fetal growth restriction (FGR), characterized by neuroinflammation and reduced blood-brain barrier integrity, which is normally maintained by the neurovascular unit. We examined whether umbilical cord blood stem cell (UCBC) treatment stabilized the neurovascular unit and reduced brain injury in preterm ventilated FGR lambs.

**Methods:**

Surgery was performed in twin-bearing pregnant ewes at 88 days’ gestation to induce FGR in one fetus. At 127 days, FGR and appropriate for gestational age (AGA) lambs were delivered, carotid artery flow probes and umbilical lines inserted, lambs intubated and commenced on gentle ventilation. Allogeneic ovine UCBCs (25 × 10^6^ cells/kg) were administered intravenously to lambs at 1 h of life. Lambs were ventilated for 24 h and then euthanized.

**Results:**

FGR (*n* = 6) and FGR+UCBC (*n* = 6) lambs were growth restricted compared to AGA (*n* = 6) and AGA+UCBC (*n* = 6) lambs (combined weight, FGR 2.3 ± 0.4 vs. AGA 3.0 ± 0.3 kg; *p* = 0.0002). UCBC therapy did not alter mean arterial blood pressure or carotid blood flow but decreased cerebrovascular resistance in FGR+UCBC lambs. Circulating TNF-α cytokine levels were lower in FGR+UCBC vs. FGR lambs (*p* < 0.05). Brain histopathology showed decreased neuroinflammation and oxidative stress, increased endothelial cell proliferation, pericyte stability, and greater integrity of the neurovascular unit in FGR+UCBC vs. FGR lambs.

**Conclusions:**

Umbilical cord blood stem cell therapy mitigates perinatal brain injury due to FGR and ventilation, and the neuroprotective benefits may be mediated by stabilization of the neurovascular unit.

## Introduction

Fetal growth restriction (FGR), due to placental insufficiency, leads to progressive reduction of oxygen and nutrient supply to the developing fetus. Perinatal brain injury due to FGR is associated with impairments in brain structure and function. The neurological outcomes associated with FGR depend on the gestation at onset of FGR, severity of growth restriction, degree of fetal cardiovascular adaptation, and gestation at birth [1, 2]. When FGR infants are delivered premature, they often require mechanical ventilatory support over the first few days of life. Mechanical ventilation can have detrimental effects on the immature preterm brain [3, 4]. In preterm FGR offspring, neonatal ventilation leads to increased risk of brain injury [5, 6], contributed by a greater susceptibility to neuroinflammation and blood-brain barrier (BBB) breakdown in FGR offspring [6].

Stem cell therapies for non-hematological indications have received much attention recently, including for perinatal neuroprotection and neuroregeneration [7–9]. Preclinical studies support that umbilical cord blood cell (UCBC) therapy decreases the progression of perinatal brain injury. UCBCs are neuroprotective for the preterm brain when administered in ovine models of hypoxia and inflammation-induced preterm brain injury [10–12]. More recently, it has been shown that a single administration of UCBC therapy resulted in improvement in long-term behavioral outcomes in a rat model of neonatal hypoxic ischemic injury [13]. Umbilical cord blood collected at birth has a high cell yield and a wide variety of stem and progenitor cells, which are shown to mediate positive benefits on glial cells, neurons, and cells that maintain the BBB [7, 14–16]. These and other studies show that the neuroprotective or neuroreparative benefits of UCBC for the immature brain acts through anti-apoptotic, anti-inflammatory, pro-angiogenic, neurogenic, anti-oxidant, and BBB protective mechanisms [9–12].

The neurovascular unit (NVU) describes the intimate cellular relationship between neurons, glia, and the neurovasculature (endothelial cells, pericytes, astrocytes) and plays a critical role in the development of brain structure and function [17]. The NVU mediates substrate supply to brain tissue, facilitates cell interactions, and provides structural integrity to the BBB. Disruption to the NVU can occur in the developing brain in response to placental insufficiency and FGR [18]. In turn, altered cell interactions lead to an increased risk of BBB breakdown and may have significant adverse implications for injury-induced angiogenesis and repair [19]. Administration of UCBC in adult stroke induces changes in the neurovasculature, resulting in reduced brain neuropathology and improved neurological outcome [20, 21]. It is unknown whether UCBC therapy mediates similar effects on the neurovascular unit in the neonatal brain.

Accordingly, this study evaluated the therapeutic potential of UCBC therapy to prevent or modulate mediators of brain injury in preterm ventilated FGR lambs. We have assessed the role of the neurovasculature in perinatal brain injury and repair and examine a therapeutic target for growth-restricted babies at risk of brain injury. We hypothesized that UCBCs would mitigate the harmful effects of neonatal ventilation on the preterm FGR lamb brain by targeting and stabilizing structural components of the NVU.

## Material and methods

### Ethics approval

Experiments complied with the National Health and Medical Research Council (NHMRC) of Australia guidelines for the care and use of animals for scientific purposes and were approved by Monash Medical Centre Animal Ethics Committee A. The experiments have been reported in compliance with the ARRIVE guidelines (Animal Research: Reporting in Vivo Experiments).

### Surgery to induce FGR and ventilation of lambs

Procedures to induce FGR and delivery followed by ventilation in preterm lambs have been previously described [6]. In brief, surgery for single umbilical artery ligation (SUAL) was performed at ~ 88 days’ gestation to induce early onset FGR in one fetus of twin-bearing Border-Leicester Merino crossbred ewes. The other fetus acted as the control. At ~ 126 days’ gestation, the pregnant ewe was anesthetized using thiobarbital followed by gas anesthesia and a cesarean section was undertaken. With the lamb exposed within the uterus but not yet delivered, a flow probe (size 4; ADInstruments, Castel Hill, Australia) was inserted around the carotid artery, and the lamb then delivered and the umbilical cord clamped and cut. The lambs were dried, weighed, and transferred to an infant warmer (Fisher and Paykel, Auckland, NZ) where each lamb was intubated (4.0-mm cuffed endotracheal tube), lung liquid passively drained, and gentle ventilation commenced. Umbilical vein and artery catheters were immediately inserted and secured using silk sutures. A pulse oximeter probe (Masimo, Irvine, CA) was placed on the lamb’s tail for the measurement of transcutaneous oxyhemoglobin saturation levels (SpO_2_). Near-infrared spectroscopy (NIRS; Fore-Sight Tissue Oximeter, CAS Medical Systems Inc., Branford, CT) was used for continuous recording of cerebral oxygenation, via placement of probes over the frontoparietal head region and covered with a lightproof dressing. Cerebral oxygenation was expressed as tissue oxygenation index (TOI, %) at 0.5 Hz [22]. Ventilation of the preterm FGR and AGA lambs was initiated using assist control ventilation (Babylog 8000+, Dräger, Lüberk, Germany) with an initial peak inspiratory pressure (PIP) of 30 cmH_2_O and positive end-expiratory pressure of 5 cmH_2_O for the first 10 min. The inspired oxygen fraction (FiO_2_) commenced at 0.3, but was adjusted to maintain SpO_2_ between 91 and 95% after initial resuscitation. All lambs received prophylactic surfactant via the endotracheal tube (100 mg kg^−1^, Curosurf; Chiesi Pharma, Parma, Italy) at 10 min after birth.

Lambs were ventilated for 24 h on volume guarantee mode with a tidal volume (*V*_T_) set at 5–7 mL kg^−1^. Throughout ventilation, lambs were sedated by continuous infusion of Alfaxan (3 mg kg^−1^ min^−1^; CenVet, Lynbrook, Australia) through the umbilical vein catheter. Lamb physiological well-being was assessed via umbilical arterial blood gas parameters measured at 1, 6, 12, and 24 h post delivery. Lambs were euthanized at 24 h by intravenous pentobarbital sodium overdose (100 mg kg^−1^ I.V.; Valabarb, Rutherford, Australia). All ventilation and physiological data was digitally acquired using Powerlab (1 kHz) and Lab Chart 8 software (ADInstruments, Castle Hill, Australia).

### Cell collection and preparation

The umbilical cord blood was collected at cesarean section from a separate cohort of healthy term lambs (144–145 days’ gestation). The umbilical cord was clamped, and the blood from the placental side was collected into heparinized syringes. UCBCs were then isolated from the buffy coat layer by centrifuging the blood at 3100 rpm for 12 min at room temperature, with no brake, and excess red blood cells removed using red blood cell lysis buffer. The cells were resuspended in fetal bovine serum with 10% DMSO (Merck, Darmstadt, Germany) and cryopreserved in liquid nitrogen. The cells were thawed immediately before administration and DMSO removed by washing cells with media (DMEM/F12, 10% FBS, 1% antibiotics). Cell yield and viability were assessed using trypan blue dye exclusion before administration. Cells were labeled with carboxyfluorescein succinimidyl ester (CFSE) to enable tracking of the cells within the brain [23]. For this study, allogeneic umbilical cord blood mononuclear cells (25 million/kg) were suspended in 2–3 ml of sterile saline and were given intravenously (via the umbilical vein) to the preterm ventilated lambs at 1 h of life.

### Brain pathology

After euthanasia, cerebrospinal fluid (CSF) was collected with an 18G needle and a 3-ml syringe and the brain removed and weighed. The left brain hemisphere was divided into four sections (frontal, middle (× 2), occipital) and frozen for analysis. The right brain hemisphere was coronally cut into 5-mm slices and fixed in formalin for 48–72 h and then embedded in paraffin (ProSci Tech, Thuringowa, Australia) for histological and immunohistochemistry analysis.

### Molecular assessment and cytokine assays

Concentrations of intracellular adhesion molecule (ICAM), vascular cell adhesion molecule (VCAM), interleukin (IL) 3, IL6, neuron-specific enolase (NSE), decorin, interferon (IFN) γ, IL17A, IL21, IL8, IP10, monocyte induced by gamma interferon (MIG), secreted frizzled-related protein (sFRP) 3, tumor necrosis factor (TNF) α, and vascular endothelial growth factor (VEGF)-A were assessed in the lamb serum and CSF samples using a human 5-plex and ovine 10-plex quantibody array following the manufacturer’s instructions (Crux Biolab, Scoresby, Australia). Ovine brain-derived neurotrophic factor (BDNF), nerve growth factor (NGF), and amyloid precursor protein (APP) ELISA assays were conducted on homogenized brain white matter tissue. Lastly, endothelin-1 receptor antibody (EDNRA) ELISA (Crux Biolab, Scoresby, Australia) was conducted using the manufacturer’s instructions on serum samples at 1, 6, and 12 h after birth.

### Immunohistochemistry

#### Single-label staining

Cerebral cellular apoptosis was assessed using activated caspase-3 (Cat# AF835, R&D Systems, Minneapolis, MN), astrocytes were assessed using glial fibrillary acidic protein (GFAP, Cat# G3893, Sigma-Aldrich, Castle Hill, Australia), inflammatory microglial cells were evaluated using ionized calcium binding adapter molecule 1 (Iba-1, Cat# 019-19741, Wako Pure Chemical Industries, Osaka, Japan), BBB permeability was assessed using albumin extravasation (Cat# S4265-2ML, Sigma-Aldrich, Castle Hill, Australia), and oxidative stress was assessed using 4-hydroxynonenal (4HNE, Cat# 393207-100ul, Merck, Germany). Ki67 (Cat# RM-9106-S, Thermo Fisher Scientific, Waltham, MA) was used to study cell proliferation, and endothelial cell proliferation was evaluated via expression of glucose transporter-1, which is present on mature endothelial cells (Glut-1; Cat# AB14683, Abcam, Melbourne, Australia). Standard immunohistochemistry protocols were followed. Briefly, for any single-label immunohistochemistry analysis, brain blocks at the level of caudo-putamen and the dorsal tegmental bundle containing the cortical gray matter (CGM), subcortical white matter (SCWM), periventricular white matter (PVWM), and hippocampus (hippo) and subventricular zone (SVZ) were sectioned at 10 μm. Serial sections were placed on SuperFrost glass slides and dewaxed in xylene followed by rehydration in serial ethanol solutions. Antigen retrieval was carried out by heating in citric acid buffer (pH 6) for 15 min (3 × 5 min) and then allowing the hot buffer to cool at room temperature for a further 20 min. Endogenous peroxidase activity was blocked by 0.3% hydrogen peroxide buffer in 50% methanol. Non-specific binding was blocked by animal serum (goat or rabbit serum in bovine serum albumin). Primary antibody was then added and sections incubated at 4 °C overnight. The following day, sections were incubated in corresponding secondary antibody followed by streptavidin horseradish peroxidase (HRP; Cat# GERPN1051-2ML, 1:200, Amersham Bioscience, UK). Staining was visualized using 3,3′-diaminobenzidine and coverslipping using mounting medium (DPX, Cat# 100579, Merck, Kilsyth, Australia).

#### Double-label staining

Double-labeled fluorescent immunohistochemistry was conducted to determine the relationship of the desmin and smooth muscle actin (α-SMA), which labels the pericytes of the neurovascular unit. After dewaxing the sections, endogenous peroxidases were blocked with 0.3% hydrogen peroxide in 50% methanol, and sections were then washed with sodium borohydride (10 mg/mL) in PBS to reduce autofluorescence. Sections were subsequently treated with serum-free protein blocker (Cat# X090930-2, DAKO Australia, Campbellfield, Australia) and incubated with mouse monoclonal α-SMA (Cat# A522-200UL, 1/50; Sigma-Aldrich, USA) and rabbit polyclonal anti-desmin (Cat# D8281-1ML, 1/50; Sigma-Aldrich, USA) to identify the degree of pericyte coverage, as previously described by our group [18]. Fluorescent secondary antibodies were used to study the interaction of the two proteins in the neurovascular unit.

### Quantitative analysis of brain injury

Sections were viewed at a magnification of × 400 using light microscopy (Olympus BX-41, Japan) with the slides coded for blind analysis. Immunoreactive cell counts were assessed in three fields of view within regions of interest on two slides per animal, to give six fields of view per region per animal, from which an average was then calculated. Manual counts of immunopositive cells expressing GFAP (astrocytes), Iba-1 (activated or amoeboid microglia), caspase-3 (cell death), 4-HNE (oxidative stress), Ki-67 (cell proliferation), and Glut-1 (endothelial cell proliferation) were undertaken.

For cell proliferation, besides manual counting of Ki-67-positive cells in brain regions, we calculated the percentage of blood vessels with Ki-67-positive cells as compared to all blood vessels. Automated counts of percent area for Glut-1 staining were also assessed, using an automated program after tracing the blood vessel using a stylus and Glut-1 immunoreactivity assessed by the area covered. Fluorescent desmin and α-SMA colocalization (using Mander’s coefficients M1 and M2) was assessed by measuring the amount of co-localization between the red and green fluorescence within blood vessels at × 400 magnification. An image processing software (ImageJ- Fiji 2.0.0, National Institutes of Health, MD) was used for automated analysis using specific macros designed by us.

### Statistics

Statistical comparisons were carried out using GraphPad Prism (GraphPad Software v7, San Diego, CA) and SPSS (v25, IBM SPSS Statistics, Armonk, NY). Data are presented as mean ± standard deviation (S.D.) or as percentage or a fraction for semi-quantitative data (blood vessel proliferation, albumin extravasation). Group data were analyzed using one-way, two-way, or three-way ANOVA or repeated measures two-way or three-way ANOVA where appropriate and post hoc multiple comparisons test. Sources of variations for ANOVA included time factor, fetal growth (FGR vs. AGA), UCBC (FGR/AGA+UCBC vs. FGR/AGA) or a combination of these. Significance was accepted at *p* < 0.05.

## Results

### Baseline characteristics

Fetal surgery was undertaken in 13 sets of twin fetal sheep; there were 2 fetal deaths, one each in FGR and AGA lamb groups. In total, 24 newborn lambs (6 in each group (AGA, FGR, AGA+UCBC, FGR+UCBC) were included in final analyses. Animal body and specific organ weights and organ/body weight ratios are presented in Table 1. All groups had an equal number of male and female lambs (3:3). Overall, FGR lambs (FGR and FGR+UCBC) weighed 23% less than AGA lambs at birth (AGA and AGA+UCBC) (2.3 ± 0.4 kg vs. 3.0 ± 0.3 kg; *p* = 0.0002). Both FGR and FGR+UCBC lambs demonstrated an increase in brain/body weight ratio compared to AGA, indicative of brain sparing. Organ weights of the liver, lung, and heart were reduced in FGR lambs compared to AGA lambs; Table 1.

**Table 1:** Body, organ weights, and organ/body weight ratios for the lamb groups

	AGA (*n* = 6)	FGR (*n* = 6)	AGA+UCBC (*n* = 6)	FGR+UCBC (*n* = 6)
Body weight (kg)	3.0 ± 0.4	2.5 ± 0.4*	3.1 ± 0.3	2.0 ± 0.2*
Brain weight (g)	43.9 ± 2.2	43.9 ± 4.7	46.4 ± 3.7	42.4 ± 3.6
Brain/body weight (g/kg)	14.8 ± 1.7	17.9 ± 2.5*	15.2 ± 1.8	20.8 ± 2.6*
Liver weight (g)	127.2 ± 26.1	94.4 ± 30.2*	121.4 ± 16.7	89.0 ± 15.3*
Liver/body weight (g/kg)	42.1 ± 3.6	37.4 ± 7.6	39.5 ± 4.5	41.1 ± 21.6
Lung weight (g)	93.5 ± 11.2	80.0 ± 37.3	95.7 ± 9.3	71.8 ± 4.2*
Lung/body weight (g/kg)	31.6 ± 5.5	31.4 ± 10.2	31.4 ± 4.6	33.3 ± 17.4
Heart weight (g)	26.7 ± 4.3	20.4 ± 6.8*	25.2 ± 2.5	16.4 ± 1.8*
Heart/body weight (g/kg)	8.9 ± 1.5	8.1 ± 1.9	8.2 ± 1.3	7.5 ± 3.9

Data expressed as mean + SD. Two-way ANOVA analysis was applied for each parameter. *Significant differences between corresponding FGR vs. AGA lamb groups

### Physiological parameters and ventilation requirements

Blood gas parameters (Additional file 1: Table S1) and ventilation parameters (Additional file 1: Table S2) are shown at 1, 6, 12, and 24 h after birth. There were no individual time point differences for any blood gas variables across the groups. Ventilation requirements were not significantly different across the four lamb groups throughout the experiment, albeit the FGR groups were more likely to be on higher FiO_2_ and have lower lung compliance at 24 h than AGA groups .

### Hemodynamics

**Fig. 1:**
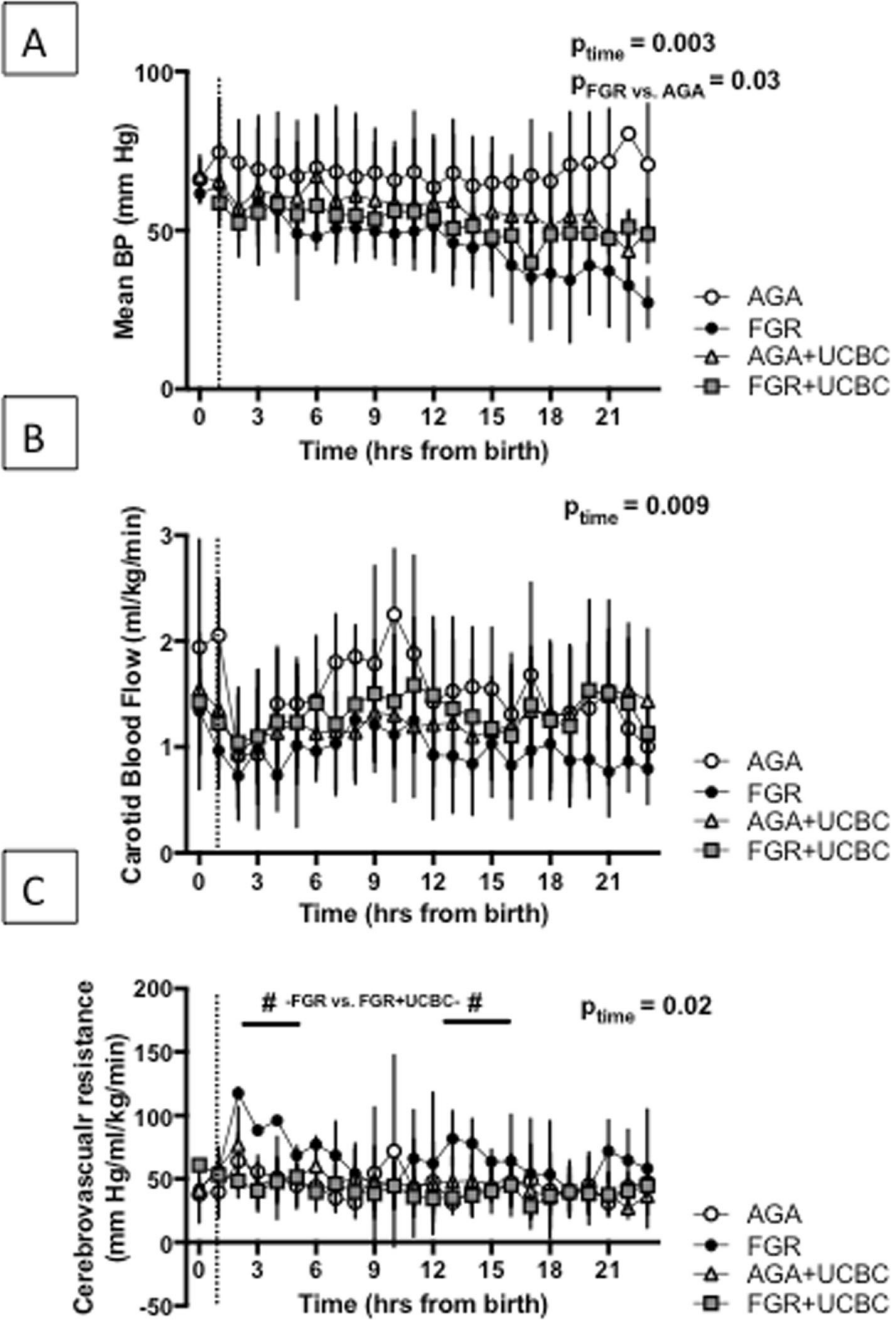
**a** Mean arterial blood pressure (MAP) across lamb groups over the course of the experiment. **b** Carotid blood flow (CBF) as a surrogate for cerebral blood flow across lamb groups. **c** Cerebrovascular resistance for lamb groups. Number sign denotes significant differences between FGR vs. FGR+UCBC group at highlighted time points, *p* < 0.05. Dotted lines denote the timing of UCBC administration; three-way RM ANOVA was applied across all parameters. *p*_time_ signifies time interaction; *p*_FGR vs. AGA_ signifies growth interaction

Figure 1 demonstrates hemodynamic measures in all groups over the 24 h of the experiment. Mean arterial blood pressure remained stable over 24 h in the AGA group. Overall however, there was a time-dependent decrease in MAP over the time course of the experiment (*p* = 0.003). The FGR groups had lower MAP than AGA groups (*p* = 0.03). UCBC treatment did not affect MAP in the AGA+UCBC or FGR+UCBC lambs. There was a significant effect of time (interaction using three-way ANOVA) across groups on carotid blood flow (*p*_time_ = 0.009), but no differences between groups or effect of UCBCs. There was a significant effect of time on cerebrovascular resistance (*p*_time_ = 0.02). Cell therapy was associated with a decrease in cerebrovascular resistance in FGR+UCBC vs. FGR lambs within 2 h after cell therapy (mean difference 69.0 mmHg/ml/kg/min (95% CI 48.9–89.1, *p* < 0.05) and from 12 to 15 h after birth (13 h, mean difference 46.4 mmHg/ml/kg/min (95% CI 1.8–91.5, *p* < 0.05). There was no change in the tissue oxygenation index as a result of cell therapy (Additional file 1: Figure S1). We also measured levels of the EDNRA in the serum as a marker of vascular reactivity; however, there was no significant change in EDNRA levels from baseline over the duration of the experiment (data not shown).

### Immunohistochemistry and molecular analysis

**Fig. 2:**
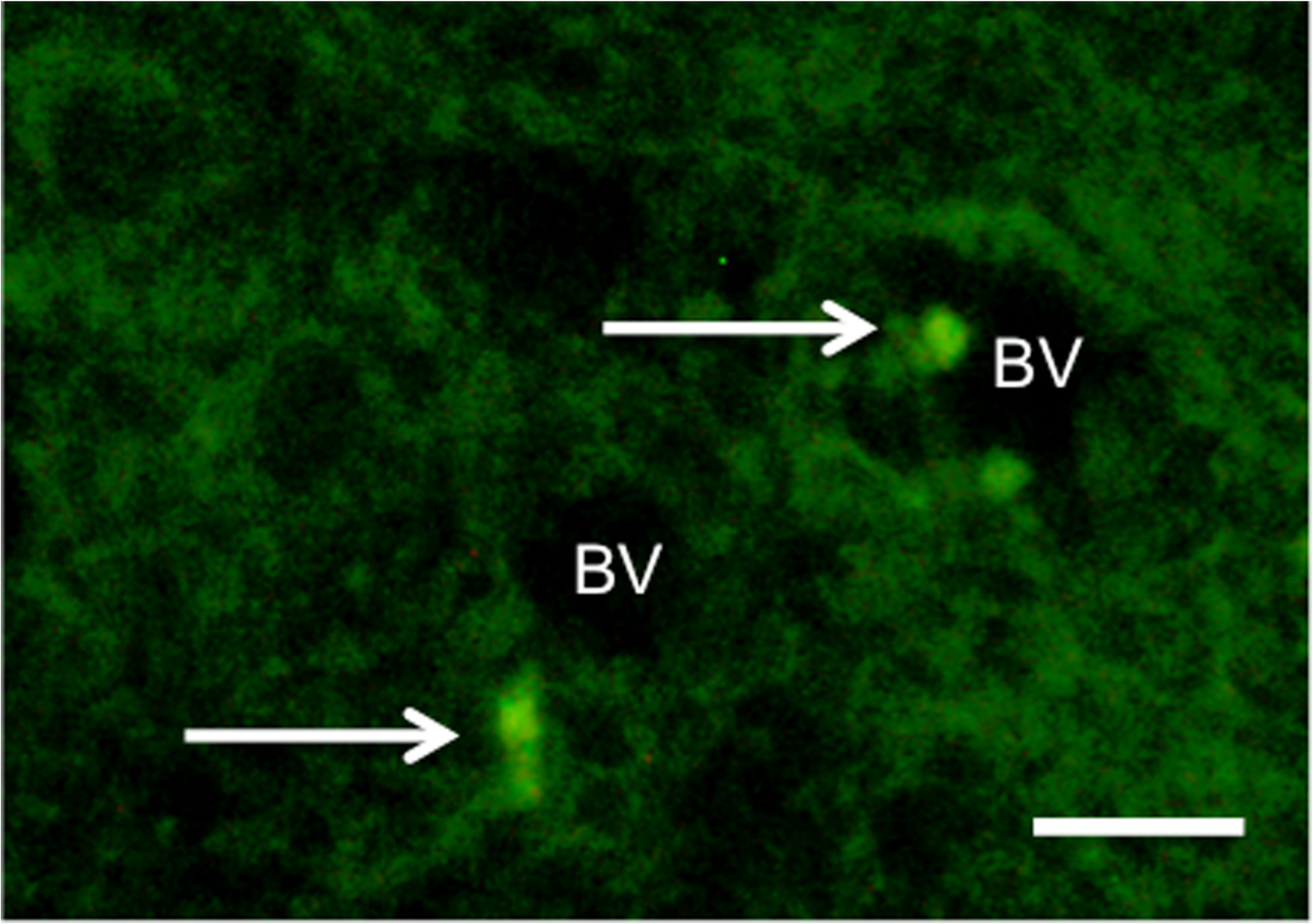
Representative photomicrograph of CFSE-labeled cells (arrows) in subcortical white matter of a FGR+UCBC lamb. BV blood vessel. Scale bar = 50 μm

**Fig. 3:**
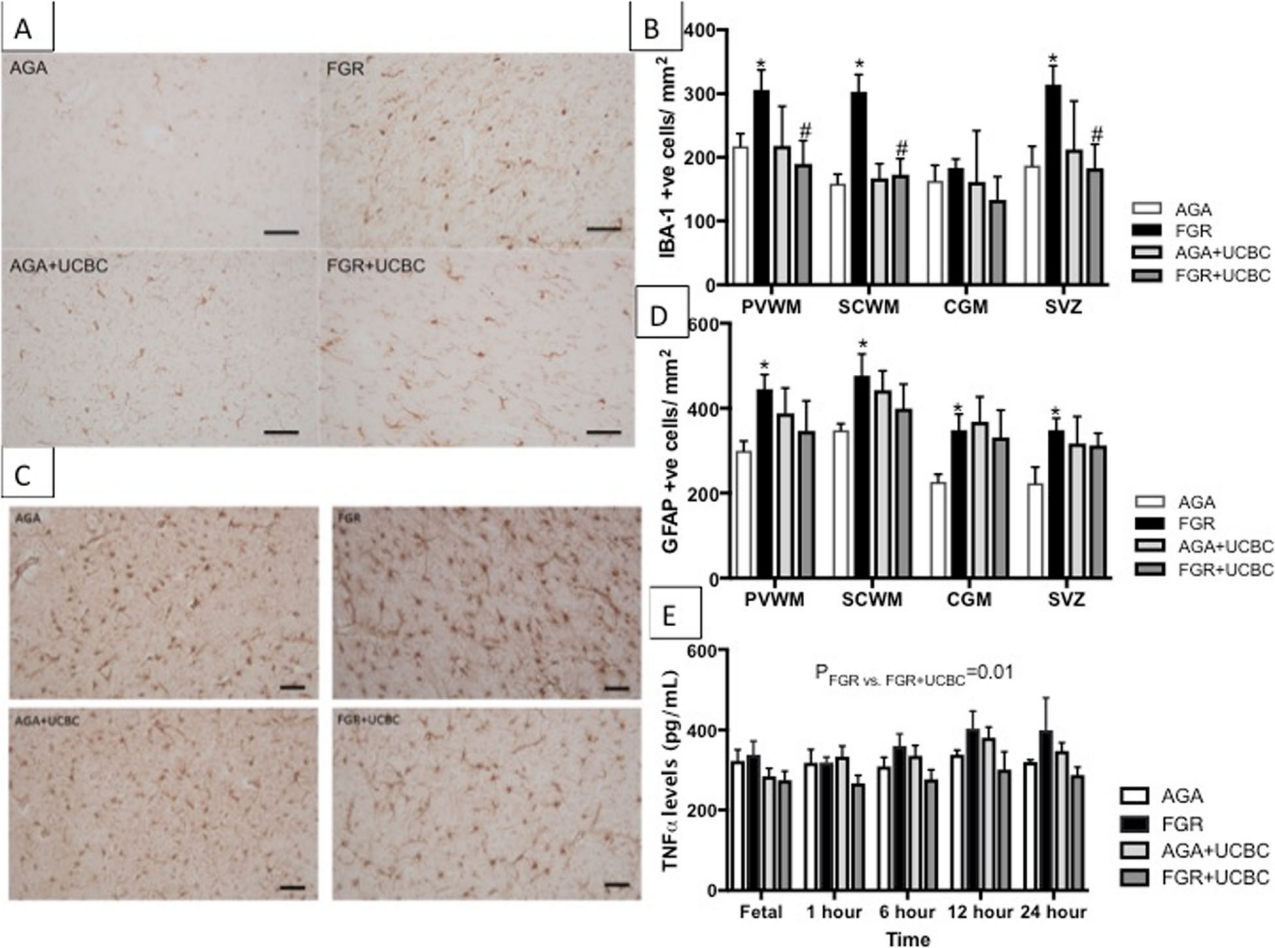
**a** Representative photomicrographs of Iba-1-positive inflammatory cells in SCWM across groups. **b** Quantitative analysis of Iba-1 cell counts across brain regions; two-way ANOVA applied. **c** Representative photomicrographs of GFAP-positive astrocytes in SCWM across groups. **d** Quantitative analysis of GFAP cells across brain regions; two-way ANOVA analysis applied. **d** Serum levels of pro-inflammatory cytokine, TNF-α across groups over the course of the experiment; two-way RM ANOVA applied. No significant rise in pro-inflammatory cytokines in FGR as compared to AGA lamb groups, but a significant decrease in TNF-α levels seen in FGR+UCBC vs. FGR. Asterisk denotes FGR significantly different to AGA. Number sign denotes FGR+UCBC significantly different to FGR; significant differences accepted at *p* < 0.05. All scale bars = 50 μm

CFSE-labeled cells were distributed diffusely in low numbers, mostly near blood vessels in the white matter of the brains of AGA+UCBC and FGR+UCBC animals (Fig. 2). We utilized two immunohistochemical markers as measures of neuroinflammation (Fig. 3), Iba-1 to assess activated microglia and GFAP to assess astrogliosis. The number of activated microglial cells was increased in FGR brains in the PVWM, SCWM, and SVZ (*p* < 0.05) compared to AGA. There was no difference in the number of activated microglia in FGR+UCBC compared to AGA across all brain regions examined, but cell counts in the FGR+UCBC group were reduced compared to FGR within the PVWM, SCWM, and SVZ (*p* < 0.05). The number of GFAP-positive astrocytes was significantly increased in the FGR animals versus AGA across all brain regions examined (*p* < 0.05), while no other group was significantly different to the AGA group. Serum TNF-α protein concentration showed a significant elevation in the FGR group above that in the FGR+UCBC group (*p* = 0.01; Fig. 3e). There were no significant differences seen in any other cytokines measured in serum or CSF (data not shown).

**Fig. 4:**
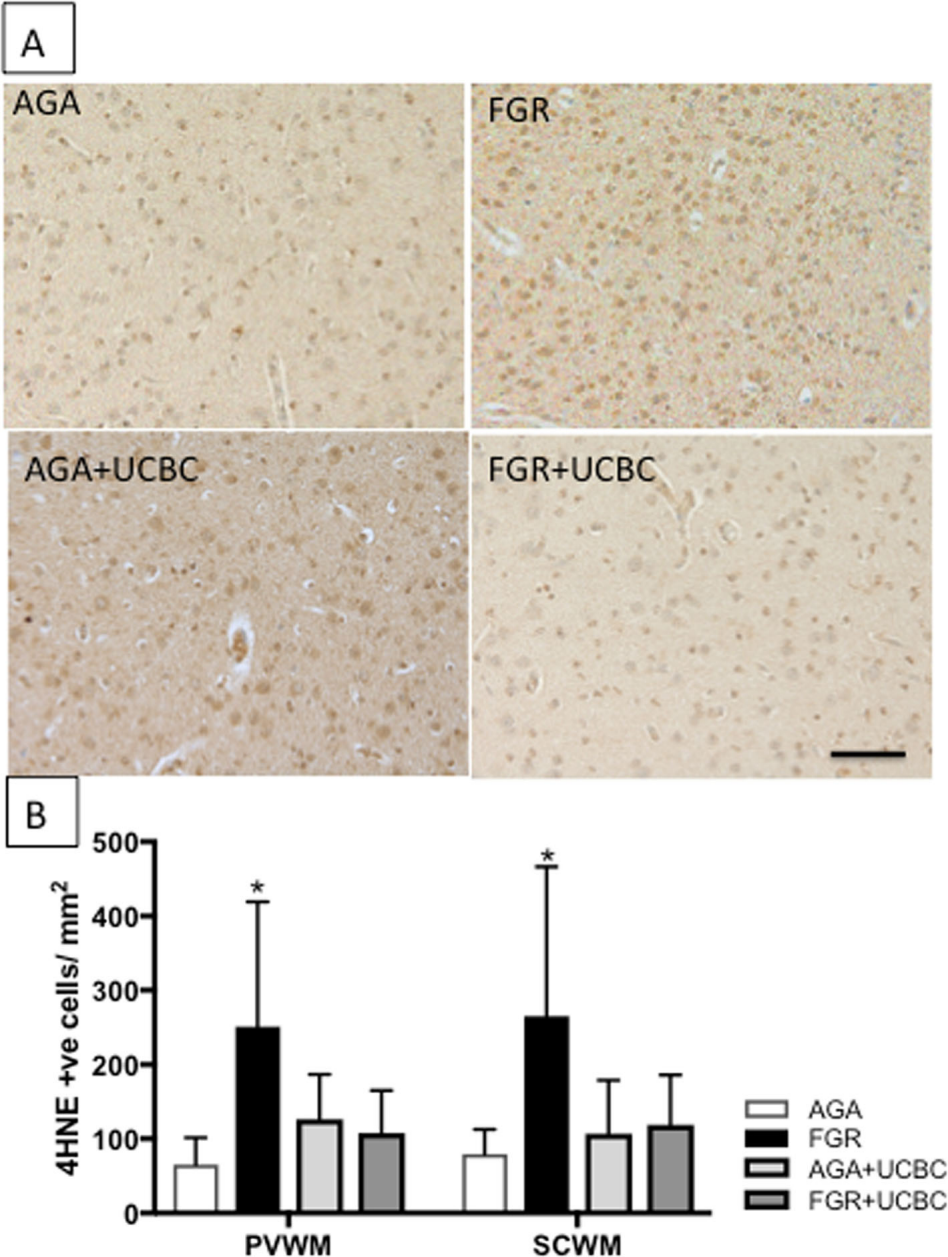
**a** Representative photomicrographs of 4-HNE-positive cells demonstrating oxidative stress in SCWM across groups. **b** Quantitative analysis of 4-HNE cell counts across white matter brain regions; two-way ANOVA applied. Asterisk denotes FGR significantly different to AGA; significant differences accepted at *p* < 0.05. All scale bars = 50 μm

We determined brain levels of oxidative stress by performing immunohistochemistry for 4-HNE in white matter brain regions (Fig. 4). We observed a significant increase in cellular oxidative stress within the PVWM and SCWM of FGR brains compared to AGA (*p* < 0.05). No other significant differences were observed in levels of cellular oxidative stress across groups. Caspase-3-mediated cell death was not significantly different across groups in brain regions examined (Additional file 1: Figure S3).

### Neurovascular unit

**Fig. 5:**
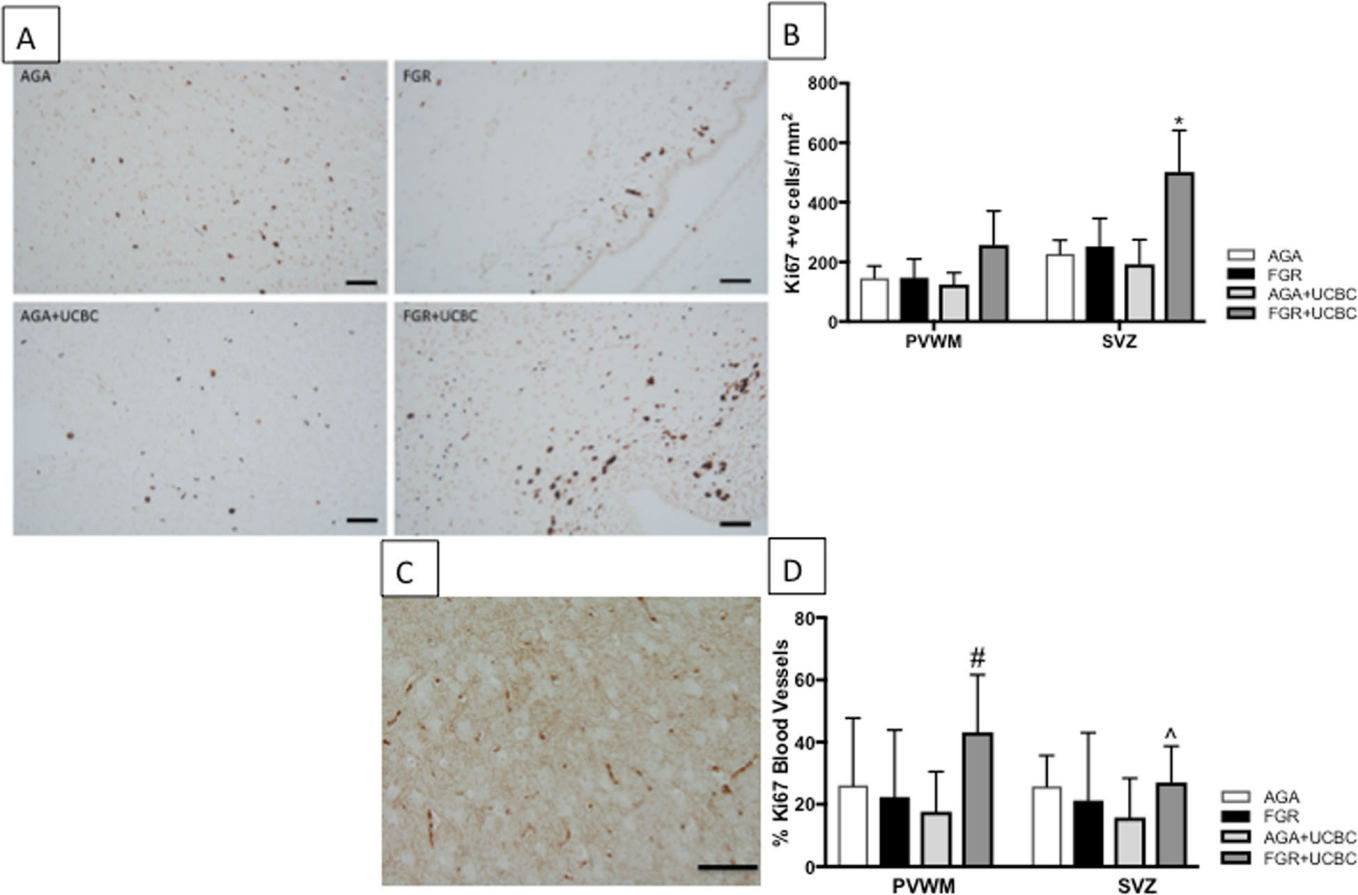
**a** Representative photomicrographs of Ki-67-positive cell proliferation in SVZ across groups. **b** Quantitative analysis of Ki-67 cell counts across brain regions; two-way ANOVA applied. **c** Representative photomicrographs of Ki-67-positive blood vessels in PVWM in FGR+UCBC animals. **d** Quantitative analysis of Ki-67-positive blood vessels across brain regions. Number sign denotes significant difference between FGR+UCBC vs. all other groups. Caret symbol denotes significant difference between FGR+UCBC vs. AGA+UCBC group. Asterisk denotes significant difference between FGR+UCBC vs. all other groups; significant differences accepted at *p* < 0.05. All scale bars = 50 μm

**Fig. 6:**
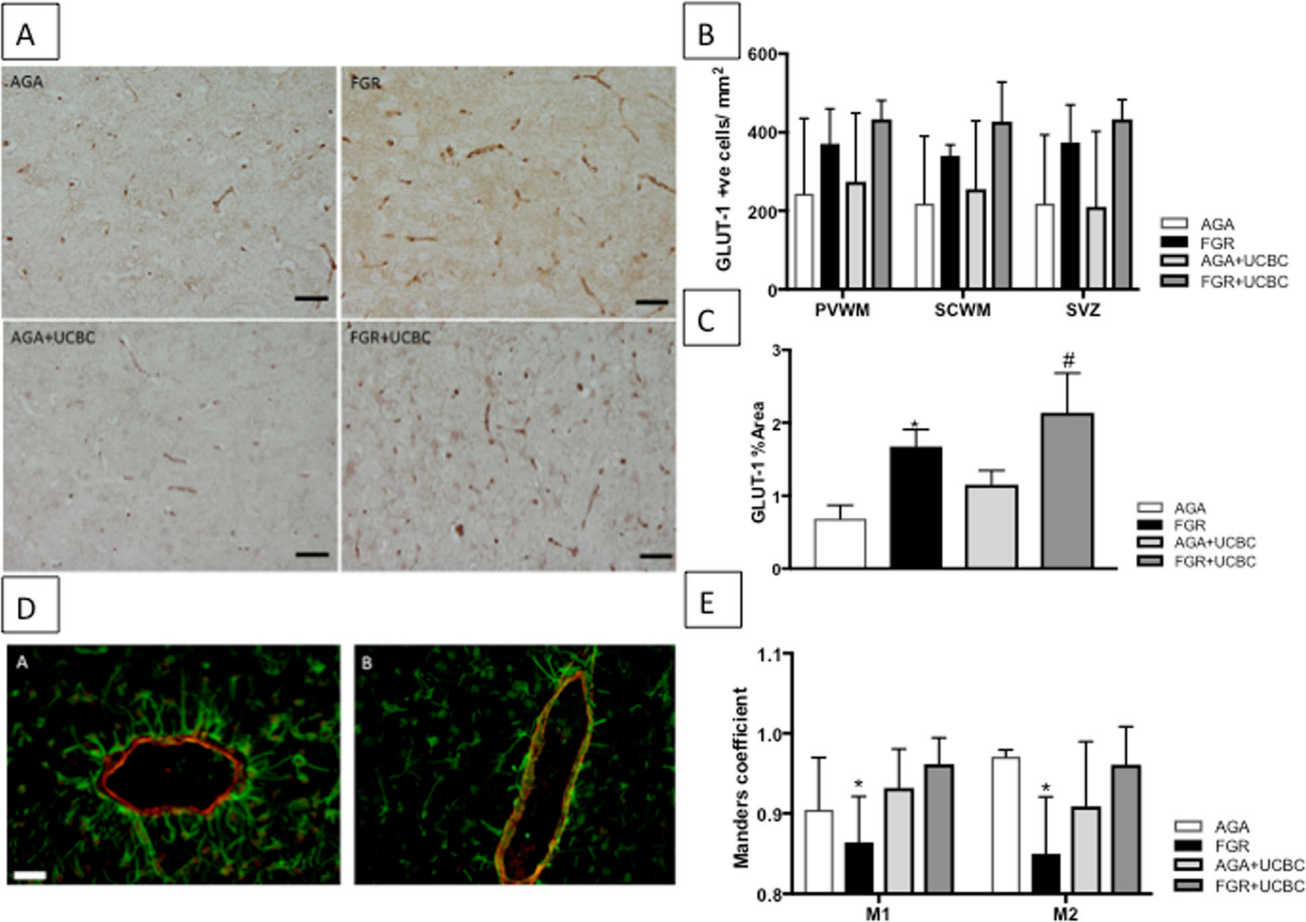
**a** Representative photomicrographs of Glut-1-positive endothelial cell proliferation in PVWM across groups. Scale bar = 50 μm. **b** Quantitative analysis of Glut-1 cell counts across brain regions; two-way ANOVA applied. **c** Quantitative analysis of Glut-1-positive percent area in PVWM across groups. **d** Representative photomicrographs of Desmin-SMA double label fluorescent staining showing poor (**a**, FGR) and good (**b**, FGR+UCBC) co-localization within the neurovascular unit. Scale bar = 100 μm. Asterisk denotes significant difference between FGR vs. AGA groups. Number sign denotes significant difference between FGR+UCBC vs. all other groups. **e** Quantitative analysis of co-localization coefficients (M1 and M2), showing significant reduction (*) in co-localization coefficients in FGR lambs, which is normalized with UCBC therapy; significant differences accepted at *p* < 0.05

Cell proliferation was examined using Ki-67 immunohistochemistry (Fig. 5a, b) and revealed a significant increase in cell proliferation in the SVZ of FGR+UCBC lamb brains. We often observed Ki-67-positive cells in association with blood vessels (Fig. 5c, d) and, therefore, next calculated the percentage of Ki-67- positive blood vessels (Ki-67 positive/total blood vessels in a field of view) in the PVWM and SVZ. There was a significant increase in Ki-67-positive blood vessels in both regions (*p* < 0.05). We next assessed whether distribution and number of endothelial cells in blood vessels was altered by FGR or FGR+UCBC by quantifying the glucose transporter (Glut-1) present in the cerebral vasculature. Both FGR and FGR+UCBC lamb brains showed an increase in endothelial cell coverage compared to AGA brains (*p* < 0.05), and the FGR+UCBC group was also significantly increased compared to all other groups (Fig. 6a–c).

Pericyte coverage of individual blood vessels was examined using desmin α-SMA double-labeled immunofluorescence (Fig. 6d). FGR lambs showed a significant decrease in co-localization as studied by Mander’s coefficient. UCBC therapy normalized co-localization in FGR+UCBC lamb brains as evidenced by no difference with pericyte coverage compared to AGA (Fig. 6e). Lastly, blood-brain barrier function was assessed using albumin immunohistochemistry. We observed that 5 of the 6 FGR lamb brains showed evidence of increased BBB dysfunction (albumin extravasation into the brain parenchyma surrounding blood vessels) as compared to 1 out of 6 AGA brains. Only 1 of the 6 FGR+UCBC brains demonstrated albumin extravasation into the brain parenchyma.

## Discussion

FGR infants are frequently delivered prematurely and being born preterm exacerbates brain injury associated with FGR [24], but the mechanism/s underlying increased neuropathology remain largely unknown. Here, we demonstrate that neuroinflammation and oxidative stress are upregulated in the brain of preterm FGR offspring that are ventilated, and we show, for the first time, that allogeneic umbilical cord blood cell therapy has neuroprotective properties for preterm FGR offspring via modulation of these aberrant responses. This is important because ventilation-induced cerebral inflammation and oxidative stress are observed more in FGR offspring and likely mediate additive damage to developing white and gray matter of the brain [2]. Further, our results demonstrate that the neuroprotective benefits of UCBC are mediated, at least in part, via stabilization of the neurovascular unit.

### Neuroinflammation

Animal models of FGR show that neuroinflammation is upregulated in areas of white and gray matter that are vulnerable to damage [25–27]. Cerebral inflammation can disrupt neuronal and oligodendrocyte development [28] leading to life-long neurological deficits. Our results demonstrate that mechanical ventilation induces an exacerbated microglial and astrocyte response in the FGR lamb brain, relative to AGA lambs. We did not include an unventilated group in this study, but we have previously shown that neuroinflammation is increased with ventilation in this preterm FGR ventilation model and other preterm lamb models [6, 29, 30]. This selective neuroinflammation in FGR lambs may be a contributing factor towards the additive neuropathology observed in FGR infants who are born preterm. Accordingly, therapeutic intervention soon after birth to reduce neuroinflammation is likely to be neuroprotective for the FGR brain.

The administration of umbilical cord blood stem cells to ventilated FGR lambs at 1 h after birth significantly decreased brain inflammation when compared to untreated ventilated FGR animals. Microglial cell activation was decreased across all white matter brain regions examined in FGR+UCBC lambs. The cerebral and systemic anti-inflammatory benefits of UCBC therapy have been reported previously in a preclinical model of preterm brain injury induced via acute hypoxia-ischemia [10]. Li et al. also showed a significant correlation between increasing density of activated microglia and loss of oligodendrocytes at 10 days after insult, indicative that neuroinflammation is strongly associated with oligodendrocyte development. Further, that study showed that early administration of UCBC resulted in reduced microgliosis and restoration of oligodendrocyte cell number at 10 days after treatment [11]. While our study was conducted over a shorter period of 24 h, these results suggest that protecting the brain against neuroinflammation would have positive benefits for white matter development.

The benefits of UCBCs may be mediated by their mixed cell population. Umbilical cord blood contains a number of stem and progenitor cells, including mesenchymal stromal cells (MSCs), T-regulatory cells (Tregs), endothelial progenitor cells (EPCs), and hematopoietic stem cells (HSCs), each with differential properties that could mediate positive effects on the brain [31]. In particular, the MSCs are postulated to modulate the anti-inflammatory effects of UCBCs, but we have shown recently that the proportion of MSCs in term derived UCBC is very low, < 0.01% [32], and therefore, it is likely that other cell types mediate the anti-inflammatory effects observed in the brain. Interestingly, in response to acute hypoxia in term-equivalent neonatal rats, the EPCs were equally effective as mixed cell UCBCs for reducing microglial activation and peripheral inflammatory cell infiltration into the brain, and the EPCs were more effective at reducing behavioral deficits [31] than UCBC. Endogenous or therapeutically administered EPCs migrate towards regions of tissue injury, where they contribute towards vascular repair and remodeling [33, 34]. Thus, in light of our observation that UCBCs reduced neuroinflammation and improved vascular stability, the mechanistic role and therapeutic potential of EPCs should be further examined.

Markers of cerebral oxidative stress were also significantly reduced within the white matter of FGR+UCBC lambs. Oxidative stress can play a major role in brain injury associated with FGR, preterm ventilation-induced brain injury, and apoptosis and cell death [4, 6, 35]. Studies of UCBC therapy have also demonstrated reduced circulating oxidative stress markers in a preterm lamb hypoxic model [10] and in response to birth asphyxia [9]. Despite this, we were not able to demonstrate a significant effect of UCBC on circulating oxidative stress markers or cell death seen in the FGR+UCBC lamb brains in this study. The previous studies of Li et al. [10] and Aridas et al. [9] induced perinatal brain injury via acute and severe hypoxia-ischemia, whereas FGR fetuses in the current study are exposed to moderate hypoxia over a chronic period, secondary to placental insufficiency. Also, the timing of the examination of the brain (24 h post cell administration) in this study might have precluded observation of changes to cell death within 24 h of birth and ventilation.

### UCBC administration and practical use

Allogeneic UCBCs were collected from healthy term sheep at cesarean delivery. We utilized allogeneic cord blood cells from term ovine pregnancies in this study for three reasons: (i) we wanted to ensure that we used healthy UCBCs and the composition of stem and progenitor cells in the cord blood is different and may be compromised in pregnancies complicated by preterm birth, chorioamnionitis, or FGR [36]; (ii) we chose to administer cells at 1 h after birth, which was feasible with allogeneic administration; and (iii) we have shown previously that UCBCs from term ovine pregnancies have a more potent anti-oxidant capacity than UCBCs from preterm cord blood [10]. The optimal type, timing, and frequency of administration of UCBC cell therapy are still to be fully elucidated [15], and in this study, we chose to use the unfractionated cord blood instead of a specific cell type found within UCBC. Cells were administered at 1 h after birth because preterm infants have usually achieved relatively stable cardiorespiratory parameters by then, and it gives us an early window of opportunity for neuroprotective therapy with the best chance to prevent systemic and cerebral inflammation in this vulnerable infant population. Previous studies show that early (12 h after insult) administration of UCBCs provides greater benefit for the preterm brain following an acute hypoxic-ischemic insult when compared to later (5 days after insult) administration [11]. In a clinical feasibility study of autologous umbilical cord blood cell therapy, Cotten and colleagues found that UCBC could be collected, processed, and infused in babies mostly within 7 h of birth [7]. Consideration of feasibility of autologous transplantation of UCBC therapy is paramount for translation of such therapies into the clinic.

We examined the distribution of UCBCs within the brain after post mortem 1 day after cell administration, and we were able to observe small numbers of diffusely distributed cells predominantly localized to the white matter. While we observed low numbers of UCBC within brain tissue, we showed significant positive effects within the brain, which together support that the systemic immunomodulatory effects of UCBC, rather than cell engraftment, principally mediate the neuroprotective benefits [31]. Indeed, non-manipulated umbilical cord blood cells are known to engraft poorly in tissues [37]. While this is a positive outcome because it suggests a lesser risk of tumor formation, there may be a need for multiple, repeat dosing of cells to maximize neuroprotective benefit [38].

### Cerebral blood flow and metabolism

We did not see significant differences in carotid blood flow (CBF; as a surrogate for cerebral blood flow) across the lamb groups during the study, although it is interesting that CBF remained highest in the AGA group and lowest in the FGR group, while CBF in the FGR+UCBC animals was midway between the two. Previous studies in rodent models of stroke report different findings on the effects of UCBCs, either having no effect or causing transient improvement in cerebral blood flow [39, 40]. In any case, we did not find that CBF was adversely affected in FGR lambs after birth, at least not during the 24 h of our study. The effect of UCBC on stabilizing cerebrovascular resistance is fascinating, as we did not observe significant independent effects of UCBC on MAP or CBF, both of which contribute to cerebrovascular resistance. It is likely that the stabilization is due to a combination of improvement in CBF as a result of stabilization of the neurovascular unit (discussed below) or improved cerebrovascular metabolism. This is the first study to report any benefits of UCBC therapy on cerebrovascular resistance.

### Neurovascular unit

The importance of the neurovascular unit and its component cells have been increasingly recognized in the regulation of health and disease [41]. The neurovascular unit is critical in maintaining the integrity of the BBB. One of the major constituents of the BBB is the endothelium lining the microvessels, whose unique features largely account for the integrity of the barrier. Glut-1 was used in this study to mark the vascular endothelium, with this marker highly expressed by brain endothelium, and it is also utilized to assess glucose transport into the brain [42]. Maintaining the BBB requires significant energy, which is obtained by the uptake of glucose by endothelial cells and, as such, the endothelial cells regulate the integrity of the BBB and transport of glucose into the brain [43]. In turn, this process mediates energy-dependent survival of other glial and neuronal cells within the CNS [44]. GLUT-1 depletion is also closely related to the pathogenesis of cerebral edema [45]. We observed a significant increase in proliferating cells in the subventricular zone and periventricular white matter, which are most likely to be endothelial cells given their location on blood vessels and observed changes in GLUT-1 expression. We hypothesize that proliferation of endothelial cells in response to UCBC administration is a positive response aimed at stabilization of the energy supply to the brain. The increase in endothelial cell coverage seen with cell therapy could indicate that the UCBCs are supporting BBB integrity, which is confirmed by the results of albumin extravasation, thereby improving vascular stability. It is well described that the role of endothelial cells and astrocytes in the regulation of cerebral blood flow is crucial, and impairment in their number and function around the blood vessels leads to changes in cerebral blood flow [46]. Whether the neurovascular unit plays a similar role in the regulation of CBF in the developing brain still needs to be confirmed [47].

Together, the pericytes and astrocytes play critical regulatory roles in the NVU, including regulating capillary hemodynamic responses, angiogenesis, and neuroinflammation and contributing to the integrity of the BBB [48]. Interestingly, cell therapy did not alter the astrogliosis observed in ventilated FGR lambs. We did, however, observe an increased co-localization/association of the smooth muscle proteins of the vascular basal lamina with the pericytes in the NVU. This association would assist in maintaining stability of the NVU and regulation of cerebral blood flow and metabolism. Improved astrocyte or pericyte attachment with blood vessels is also likely to stabilize the BBB and decrease permeability to circulating proteins, as evidenced by reduced albumin extravasation around white matter blood vessels in FGR+UCBC brains.

### Overall effects

**Fig. 7:**
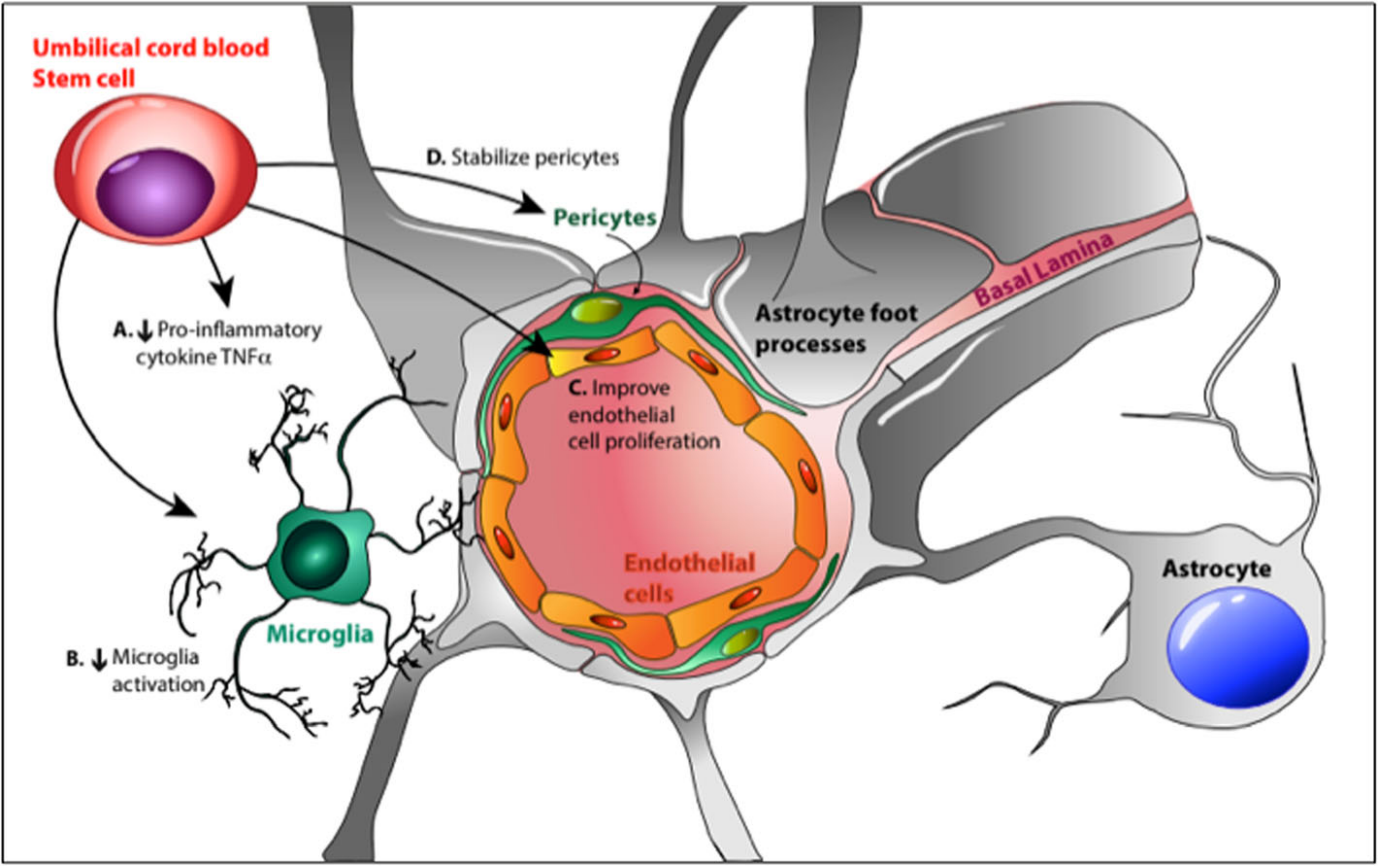
Schematic showing the possible mechanisms of action of UCBC therapy on the neurovascular unit in FGR lambs. Image courtesy: Dr. Jean Tan

The overall effects of cell therapy on the neurovascular unit of FGR lambs are summarized in Fig. 7. We demonstrate that early administration of UCBC to the FGR lamb reduces injurious processes following ventilation onset. UCBC administration provides cerebrovascular stability via a decrease in inflammation, mediated by (a) systemic decrease in pro-inflammatory factors coupled with (b) dampening of the cellular inflammatory activation of microglia. There may be some beneficial effects also on oxidative stress and stabilization of the BBB of the vulnerable FGR lamb group by (c) increasing endothelial cell proliferation and (d) increased pericyte co-localization and stability.

### Limitations

We acknowledge that there are limitations to this study, most notably that the lambs were studied for only 24 h after birth, which is a relatively short period in the time course of the developing neuropathology, and lambs were not subjected to other interventions that preterm infants may experience in neonatal care, including caffeine or inotrope administration. This period of 24 h would not be sufficient to observe the full development of brain injury, although it well described that cerebral inflammation and oxidative stress are pathogenic in the immature brain [6, 49]. It is extremely challenging to care for preterm neonatal lambs, especially growth-restricted lambs that require intensive neonatal care. Our results do however demonstrate that even in this short time frame of 24 h, significant differences were present between groups, and that indices of cerebral inflammation, oxidative stress, and neurovascular structure of animals treated with UCBC were improved compared to non-treated animals. Future studies should be designed to examine whether the short-term positive benefits of UCBC on the NVU translate to a longer-term (days to weeks) decrease in the progression of white and gray matter neuropathology. We utilized ovine UCBCs because we wished to examine the effects of allogeneic cell administration; however, the use of ovine cells precludes characterization of subsets of stem and progenitor cells of interest [10]. Finally, while we were particularly interested in the effects of UCBC on the brain, FGR infants are at risk for other neonatal morbidities, particularly related to cardiovascular and respiratory functions [2], and the effects of UCBC on these systems have not been examined as part of this study.

### Potential and impact

The results of this study are most encouraging with respect to potential clinical translation, and this is the first study to demonstrate that UCBCs are neuroprotective for the brain of preterm growth-restricted offspring. It is well described that early onset and severe (less than third centile for weight) FGR infants born preterm are at greatest risk for long-term neurodevelopmental deficits [24]. There are no neuroprotective or neuroreparative treatments that are currently offered to severe early onset FGR infants after preterm birth, but neonatal cord blood stem cell therapy is already being trialed in preterm and term brain injury in neonates showing feasibility and safety [7, 50]. We now propose that the results of this study lay the foundation for a novel therapeutic option using cord blood stem cells as an early intervention therapy for FGR infants.

## Conclusions

We have previously shown that brain inflammation and oxidative stress are significantly upregulated in the FGR brain relative to the appropriately grown brain, at 24 h after birth following mechanical ventilation. Umbilical cord blood cell therapy in the early neonatal period reduces neuroinflammation and oxidative stress in FGR lambs in response to neonatal ventilation. Our results demonstrate that neuroprotective benefits of UCBCs are mediated, at least in part, by stabilization of the neurovascular unit with the FGR brain. These results are a foundation step towards a novel neuroprotective therapy with cord blood stem cells that could be applied in the neonatal period to infants diagnosed with perinatal brain injuries like severe early onset fetal growth restriction.

## Supplementary information

**Additional file 1: Table S1.** Blood gas parameters during the course of the experiments across the lamb groups. **Table S2.** Ventilation parameters during the course of the experiments across the lamb groups. **Figure S1.** Tissue oxygenation index as measured (mean + SD) in the lamb groups across the duration of the experiment. **Figure S2.** Left panel: Representative photomicrographs of cell death seen as apoptotic cells (Caspase-3) in SCWM in lamb groups. Scale bar = 50μm. Right panel: Quantitative analysis (mean + SD) of Caspase-3 cells across brain regions. 2-way ANOVA analysis applied. No significant differences seen with cell therapy.

## Data Availability

The data that support the findings of this study are available on request from the corresponding author.

## Abbreviations

AGA: Appropriate for gestational age
BBB: Blood-brain barrier
CBF: Cerebral blood flow
CGM: Cortical gray matter
EPC: Endothelial progenitor cell
FGR: Fetal growth restriction
GFAP: Glial fibrillary acidic protein
GLUT: Glucose transporter
HNE: Hydoxynonenal
HSC: Hematopoietic stem cell
MAP: Mean arterial pressure
MSC: Mesenchymal stromal cell
NVU: Neurovascular unit
PVWM: Periventricular white matter
SCWM: Subcortical white matter
SMA: Smooth muscle actin
SVZ: Subventricular zone
UCBC: Umbilical cord blood cell
