# Supplementary material for: Neurovascular effects of umbilical cord blood-derived stem cells in growth-restricted newborn lambs: UCBCs for perinatal brain injury
Source: Stem Cell Res Ther. 2020 Jan 8;11:17. doi: 10.1186/s13287-019-1526-0 (PMC6947982; doi:10.1186/s13287-019-1526-0)
Supplement: Supplementary file 1 — Additional file 1: Table S1. Blood gas parameters during the course of the experiments across the lamb groups. Table S2. Ventilation parameters during the course of the experiments across the lamb groups. Figure S1. Tissue oxygenation index as measured (mean + SD) in the lamb groups across the duration of the experiment. Figure S2. Left panel: Representative photomicrographs of cell death seen as apoptotic cells (Caspase-3) in SCWM in lamb groups. Scale bar = 50μm. Right panel: Quantitative analysis (mean + SD) of Caspase-3 cells across brain regions. 2-way ANOVA analysis applied. No significant differences seen with cell therapy. [file 13287_2019_1526_MOESM1_ESM.docx]

**SUPPLEMENTARY FILE**

**Supplementary Table 1. Blood gas parameters during the course of the experiments across the lamb groups**

|  | | **1 hour** | **6 hours** | **12 hours** | **24 hours** |
| --- | --- | --- | --- | --- | --- |
| **pH** | **AGA** | 7.30+0.03 | 7.45+0.05 | 7.35+0.1 | 7.36+0.19 |
|  | **FGR** | 7.27+0.08 | 7.47+0.08 | 7.37+0.1 | 7.33+0.19 |
|  | **AGA+UCBC** | 7.14+0.10 | 7.39+0.02 | 7.37+0.05 | 7.34+0.06 |
|  | **FGR+UCBC** | 7.25+0.15 | 7.33+0.10 | 7.29+0.12 | 7.21+0.19 |
| **pO_2_ (mmHg)** | **AGA** | 38.0+11.8 | 47.3+11.9 | 34.6+7.1 | 39.0+7.9 |
|  | **FGR** | 35.3+7.2 | 34.9+4.1 | 38.2+9.4 | 41.8+13.5 |
|  | **AGA+UCBC** | 37.0+5.3 | 45.4+12.3 | 43.7+9.2 | 40.0+5.7 |
|  | **FGR+UCBC** | 30.4+5.8 | 41.3+13.0 | 42.2+10.7 | 35.3+9.1 |
| **pCO_2_ (mmHg)** | **AGA** | 51.3+8.9 | 39.8+5.5 | 58.7+15.8 | 45.6+15.2 |
|  | **FGR** | 52.2+6.6 | 35.1+8.6 | 49.9+7.8 | 37.6+14.6 |
|  | **AGA+UCBC** | 58.1+15.0 | 44.8+2.7 | 52.0+7.2 | 54.4+10.6 |
|  | **FGR+UCBC** | 56.1+15.3 | 53.7+15.0 | 59.5+17.3 | 62.3+17.0 |
| **Lactate (mmol/L)** | **AGA** | 3.5+0.6 | 2.1+0.7 | 1.6+0.4 | 1.4+7.1 |
|  | **FGR** | 4.2+1.3 | 3.0+0.5 | 1.8+0.3 | 1.8+3.4 |
|  | **AGA+UCBC** | 4.6+1.8 | 1.7+0.2 | 1.4+0.3 | 1.3+0.4 |
|  | **FGR+UCBC** | 4.0+1.9 | 1.9+0.4 | 1.6+0.5 | 2.6+3.1 |
| **HCO_3_^-^ (mmol/L)** | **AGA** | 27.9+4.0 | 27.5+1.4 | 31.2+2.8 | 24.3+10.4 |
|  | **FGR** | 23.6+5.1 | 25.5+3.1 | 27.4+3.3 | 15.9+12.4 |
|  | **AGA+UCBC** | 20.0+4.5 | 26.8+1.9 | 28.6+1.6 | 27.5+4.2 |
|  | **FGR+UCBC** | 24.8+3.7 | 26.9+5.1 | 28.1+5.5 | 26.3+6.3 |

Data expressed as mean+SD. No significant differences seen between any lamb groups.

**Supplementary Table 2. Ventilation parameters during the course of the experiments across the lamb groups**

|  | | **1 hour** | **6 hours** | **12 hours** | **24 hours** |
| --- | --- | --- | --- | --- | --- |
| **Compliance (ml/g/cm H_2_O)** | **AGA** | 0.016+0.020 | 0.016+0.020 | 0.018+0.008 | 0.020+0.013 |
|  | **FGR** | 0.011+0.003 | 0.016+0.000 | 0.011+0.001 | 0.008+0.001 |
|  | **AGA + UCBC** | 0.013+0.001 | 0.020+0.001 | 0.016+0.001 | 0.013+0.003 |
|  | **FGR + UCBC** | 0.014+0.002 | 0.016+0.003 | 0.016+0.002 | 0.010+0.003 |
| **Peak pressure (cm H_2_O)** | **AGA** | 16.8+1.6 | 21.0+1.6 | 21.0+2.6 | 25.0+3.4 |
|  | **FGR** | 18.6+6.4 | 20.0+3.6 | 19.6+3.4 | 26.0+6.4 |
|  | **AGA + UCBC** | 21.6+1.7 | 15.8+1.3 | 15.6+0.8 | 21.7+1.9 |
|  | **FGR + UCBC** | 18.8+1.5 | 19.2+1.4 | 20.5+2.0 | 24.7+1.7 |
| **Tidal volume (ml/kg)** | **AGA** | 5.2+0.3 | 4.9+0.3 | 5.0+0.3 | 5.6+0.5 |
|  | **FGR** | 5.2+0.4 | 5.0+0.1 | 4.6+0.3 | 4.8+1.0 |
|  | **AGA + UCBC** | 4.9+0.0 | 4.9+0.0 | 4.9+0.1 | 4.9+0.1 |
|  | **FGR + UCBC** | 5.0+0.0 | 5.0+0.0 | 4.9+0.1 | 4.9+0.1 |
| **FiO_2_** | **AGA** | 40.0+11.2 | 22.6+1.0 | 26.0+5.0 | 31.0+10.0 |
|  | **FGR** | 36.5+10.5 | 22.0+1.2 | 27.6+4.0 | 52.8+15.0 |
|  | **AGA + UCBC** | 43.1+6.9 | 25.8+4.8 | 21.0+0.0 | 24.0+1.2 |
|  | **FGR + UCBC** | 35.4+6.8 | 29.5+2.8 | 30.5+3.7 | 48.4+13.4 |

Data expressed as mean+SD. No significant differences seen between any lamb groups.

**Supplementary Figure 1.** Tissue oxygenation index as measured (mean+SD) in the lamb groups across the duration of the experiment.


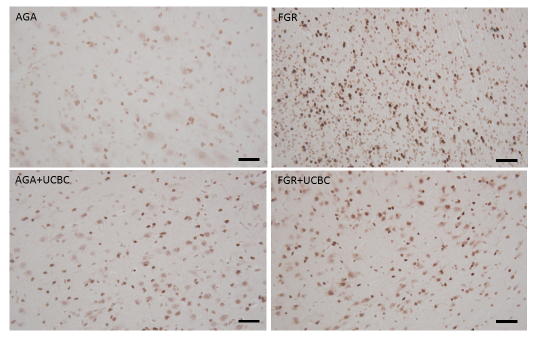


**Supplementary Figure 2.** Left panel: Representative photomicrographs of cell death seen as apoptotic cells (Caspase-3) in SCWM in lamb groups. Scale bar=50um. Right panel: Quantitative analysis (mean+SD) of Caspase-3 cells across brain regions. 2-way ANOVA analysis applied. No significant differences seen with cell therapy.
